# A single oral dose of a polyglucosamine influences the bioavailability of [9-^14^C]-Oleic acid in adult female Göttingen minipigs

**DOI:** 10.1186/s40608-016-0096-2

**Published:** 2016-03-15

**Authors:** Nicole H. P. Cnubben, Shanti L. Tel, Marleen A. Hemmes, Astrid Langenkamp-Brand, Dimitri Grossouw, Harm T. Jansen, Bert T. H. J. de Bie

**Affiliations:** TNO Triskelion BV, P.O. Box 844, Utrechtseweg 48, 3704 HE Zeist, The Netherlands; TNO, P.O. Box 360, Utrechtseweg 48, 3704 HE Zeist, The Netherlands

**Keywords:** Polyglucosamine, Chitosan, Formoline L112, Weight management, Obesity, Fat binding

## Abstract

**Background:**

Worldwide obesity has nearly doubled since 1980 and is a leading risk for global deaths, profoundly affecting morbidity, mortality, health-care costs, and professional and personal quality of life. Treatment of obesity and its consequences include lifestyle intervention, pharmacotherapy, and bariatric surgery. Polyglucosamines have been proposed as an alternative strategy for treating obesity, by reducing the amount of absorbed fat through interaction with dietary fat through various mechanisms. The objective of this study is to investigate the influence of polyglucosamine on the bioavailability of the model compound [9-^14^C]-oleic acid in female Göttingen minipigs.

**Method:**

The study consisted of two treatment groups, each consisting of six adult female Göttingen minipigs with a catheterized vena jugularis to enable frequent blood sampling. One group served as the untreated group (control) and the other group was pre-treated with 2 tablets of 500 mg formoline L112. After 30 min, all animals were dosed orally with [9-^14^C]-oleic acid. Excreta and blood samples were collected for analysis of radioactivity from 48 h pre-dose up to 144 h post-dosing. At sacrifice, the liver and contents of the gastrointestinal tract were collected for radioanalysis.

**Results:**

Upon treatment with polyglucosamine (formoline L112), the T_max_ of [^14^C]-oleic acid in plasma was shifted from 4 to 16 h, and the C_max_ decreased significantly from 14.1 μg/g to 3.3 μg/g. In addition, upon treatment with polyglucosamine the internal exposure to [^14^C]-oleic acid as reflected by the area under the curve during the 0–12 h post-dose time interval (AUC_0-12h_), is significantly decreased to 32.9 % of the plasma value of [^14^C]-oleic acid in untreated animals. Even up to 24 h post-dose, the AUC_0-24h_ is significantly decreased to 50.7 % of the plasma value in untreated animals and this significant effect is prolonged up to 60 h post-dose.

**Conclusions:**

This study shows that treatment with polyglucosamine (formoline L112) reduces (as judged by C_max_ & AUC) and delays (as judged by T_max_) fat absorption from the gastrointestinal tract into the systemic circulation and limits peak exposure to free fatty acids which may contribute to a more beneficial condition in overweight humans.

## Background

Worldwide obesity has nearly doubled since 1980. In 2008, more than 1.4 billion adults, 20 and older, were overweight. Of these, over 200 million men and nearly 300 million women were obese. About 65 % of the world’s population lives in countries where overweight and obesity kills more people than underweight. Overweight and obesity are leading risks for global deaths. Around 3.4 million adults die each year as a result of being overweight or obese. In addition, 44 % of the diabetes burden, 23 % of the ischemic heart disease burden and between 7 and 41 % of certain cancer burdens are attributable to overweight and obesity [1]. Obesity profoundly affects morbidity, mortality, health-care costs, and professional and personal quality of life [2].

The current strategies to reduce the burden of this disease and its consequences include lifestyle intervention (diet/physical activity), pharmacotherapy, and bariatric surgery. The strict requirements with respect to efficacy and safety have resulted in very limited pharmacotherapeutic options. As of September 2013, only three drugs are approved by the FDA as adjunctive therapy for chronic weight management: 1) the intestinal lipase inhibitor orlistat, 2) lorcaserin acting via the serotonergic system and 3) a phentermine/topiramate extended-release formulation, acting via modulation of catecholamines in the satiety centers of the hypothalamus, reducing appetite (phentermine) and blocking voltage-dependent sodium channels, glutamate receptors, carbonic anhydrase, and augmenting GABA activity (topiramate) [2–4]. Still there exist potentially hazardous effects as well as certain unpleasant side-effects. The side-effects resulting from orlistat’s mode of action include oily spotting, liquid stools, fecal urgency or incontinence, flatulence, and abdominal cramping [5]. Major concerns on lorcaserin include cardiovascular events, cognitive impairment, and psychiatric disorders [2, 4]. Approval of the phentermine/topiramate cocktail was denied by European regulatory authorities, who cited potential risk to the heart and blood vessels, psychiatric side effects, and cognitive side effects in explaining their decision [6].

At present, because of dissatisfaction with high costs and potentially hazardous and/or unpleasant side-effects, the potential of natural products for treating obesity is under exploration, and this may be an excellent alternative strategy for treating obesity [3]. There are numerous nutraceutical products to consider incorporating into a weight management program. Among soluble fibers, one such polysaccharide is chitosan [7]. Chitosan has the potential to be a safe compound for oral use [8, 9]. The FDA has judged Chitosan a GRAS (Generally Recognized As Safe). Chitosan polymers are not absorbed by the GIT and are unlikely to show biodistribution [9]. Chitosan has been promoted as a type of dietary fiber that may help reduce the absorption of fat due to its ability to “bind” fat and bile salts in the human gastrointestinal tract and pass this complexed fat-chitosan mixture out the body. This action reduces absorbed fat, and has prompted the use of these substances to help with weight loss [7].

The hypocholesterolemic and lipid lowering effects of chitosan are well recognized, but the specific mechanisms are not fully understood. The activity of polyglucosamine has been related to its positively charged amino-groups resulting in binding to free fatty acids (released from consumed fat) and bile salt components which results in disrupted lipid absorption in the gut. It has been suggested that polyglucosamine dissolves in the stomach, emulsifying fat and forming a gel in the intestine which entraps fat and prevents intestinal absorption. More recently, it has been proposed that polyglucosamine forms a flocculus in the duodenum which entraps dietary oil [8, 10, 11]. In addition, it has been suggested by in vitro experiments that chitosan might act as a competitive inhibitor of pancreatic lipase [12]. Overall, the mechanisms involved in chitosan’s interaction with dietary fat involve a combination of the above stated mechanisms, but none specifically have been confirmed [7, 13, 14].

Whether chitosan is actually clinically effective in cholesterol control or weight loss remains controversial. Various studies indicate that chitosan dietary supplementation had no effect on fat absorption or plasma cholesterol levels and had only a minimal, clinically insignificant, effect on bodyweight [15–19]. Other studies, however, have reported reduced blood cholesterol, lowered weight, a reduced percentage of fat and fat mass in the clinic upon chitosan supplementation [20–24]. In addition, it has been shown that 3-months administration of chitosan significantly increased insulin sensitivity in obese patients and expressed a highly notable decrease in bodyweight and triglyceride levels [25].

Formoline L112 is a linear polysaccharide composed of randomly distributed β-(1–4)-linked D-glucosamine (deacetylated unit) and N-acetyl-D-glucosamine (acetylated unit) from crustacean shells. Therefore, the formoline L112 is one dedicated specification of low molecular weight chitosan and due to its high degree of deacetylation it is part of the subgroup of polyglucosamines [13, 14]. Formoline L112 is registered as a class III medical device in Germany, and is used to limit fat absorption. The extremely high fat binding capacity of polyglucosamine has been demonstrated in vitro mimicking the environment of the gastrointestinal tract. One gram of formoline L112 has been demonstrated to bind about 700 g of dietary fat [7]. Formoline L112 is used for weight management, weight loss and to lower cholesterol blood levels. The efficacy of polyglucosamine formoline L112 for weight loss was confirmed in a randomized double-blind, placebo-controlled clinical investigation [24].

Although formoline L112 has been demonstrated to bind large amounts of dietary fats in vitro, the impact of polyglucosamine on the availability and uptake of lipids in the in vivo situation remains to be established.

The objective of this study is to provide data on the influence of formoline L112 on the bioavailability of [9-^14^C]-oleic acid (CH_3_(CH_2_)_7_CH = CH(CH_2_)_7_COOH) as model compound. In chemical terms, oleic acid is classified as a mono-unsaturated omega-9 fatty acid. The model compound oleic acid is a fatty acid that occurs naturally in various animal and vegetable fats and oils. Triglycerides of oleic acid compose the majority of olive oil. Oleic acid is the most abundant fatty acid in human adipose tissue [26]. Oleic acid is an unsaturated fatty acid which could easily be labelled on a specific position in the molecule which allows a good tracing of the active compound.

The minipig was selected to explore the effects of the fat binding polyglucosamine on the bioavailability of the model compound oleic acid. This is based on the knowledge that various anatomical and physiological aspects of (mini)pigs resemble more the human situation than other animal species. The minipig is emerging rapidly as a biomedical model for energy metabolism and obesity in humans, because it is devoid of brown fat postnatally and because of their similar metabolic features, cardiovascular systems, and proportional organ sizes [27, 28]. Both humans and (mini)pigs are true omnivores. This is reflected in the anatomy, physiology and function of the gastrointestinal system, and despite some anatomical differences in the (mini)pig the physiology of digestion remains similar to humans [29–31]. The small intestine is long, with a transit time and pH profile very similar to humans. In a recent study the mean retention time in the small intestine for 30 kg pigs was estimated to be 4 h [32]. Enzyme activity in the gut and absorption show many similarities [33]. The bioavailability of orally administered drugs that are influenced by pH or transit time can be expected to be comparable in humans and (mini)pigs. Moreover, from information in the public domain as well as publications from the European Medicines Agency (EMA) and the FDA, it is clear that the minipig is fully recognized and accepted by regulatory authorities worldwide [33]. The minipig has a significant advantage over the pig because of the reduced size, thus reducing the amount of radiolabelled compound needed for the experiments and allowing easier handling, especially as adult animals are required [33]. For the present study, adult minipigs are preferred as in younger animals there is no fat storage within the first year, since they will metabolize all available energy for growing and they will not become obese.

## Methods

### Materials

Oleic acid (purity 99 %) was purchased from Sigma-Aldrich Chemie GmbH (Germany). [9-^14^C] Oleic acid was custom synthesized at Moravek Biochemicals Inc. (US). The specific activity was 19.5 mCi/mmol and purity was 99.7 %. Polyglucosamine (formoline L112 (C_12_H_24_N_2_O_9_ (C_6_H_11_NO_4_)_n_(C_7_H_13_NO_3_)_n_), tablets of 500 mg) was obtained from Certmedica International GmbH (Germany).

### Animal model

The study was conducted with 12 female Göttingen Minipigs aged approximately 13–14 months. The welfare of the animals was maintained in accordance with the general principles governing the use of animals in experiments of the European Communities (Directive 86/609/EEC) and Dutch legislation (The Experiments on Animals Act, 1997). This includes approval of the study by TNO’s independent ethical review committee (DEC nr. 3329). The minipigs were obtained from Ellegaard Göttingen Minipigs A/S, Dalmose (Denmark). Upon arrival, the minipigs were checked for overt signs of ill health and anomalies. The animals were kept in indoor pens, according to European guidelines for housing of laboratory minipigs. The age of the minipigs at start of the study was approximately 14 months. At the commencement of the study, the weight variation of the animals did not exceed ± 20 % of the average weight.

A cannula was placed in the vena jugularis, while the animals were under anaesthesia. Animals received 13 mg/kg ketamine, 0.7 mg/kg midazolam, 0.02 mg/kg atropine and 4 mg/kg thiopental as premedication and amoxycilline/clavulanic acid as antibiotic treatment with depomycine around the catheter area.

Following cannulation, the animals were acclimatized to the laboratory conditions for at least 14 days. The minipigs were randomly allocated to a study treatment, with randomization restricted to bodyweight.

Each study group was housed in one pen. During the acclimatization period, the minipigs were socialized and trained prior to the study in order to minimalize stress and obtain collaborative animals. The animals were housed under conventional conditions in indoor pens on straw bedding with a playing ball during acclimatization. Animals were acclimatized to the stainless steel metabolism cages under test conditions, 48 h prior to the experimental start date (dosing). The room was ventilated with approximately 10 air changes per hour and targeted at a temperature of 20–24 °C with a relative humidity of 45–70 %. Lighting was artificial with a sequence of 12 h light and 12 h dark. Animals were checked twice daily for signs of ill health.

The animals were fed a commercial diet (SMP (E) SQC) (SDS Special Diets Services, Whitham, England) and received a measured amount of food (225 gr per meal) twice daily (in the morning and in the afternoon/early evening (at dosing day; about 8 h post-dosing). Fourteen days prior to the experimental start date (dosing), the standard diet was supplemented with approximately 10 g of olive oil per meal. Drinking water was supplied by N.V. Vitens Midden-Nederland and offered *ad libitum* at all times.

### Study design

The study consisted of two treatment groups, each consisting of six female Göttingen minipigs. Group A served as the untreated group (control) and group B was pre-treated with polyglucosamine as follows; the animals of group B were dosed with 2 tablets of 500 mg formoline L112 (1 g formoline L112 in total) followed by approximately 10 ml of water via a syringe. The 2 tablets of polyglucosamine were inserted into 2 gelatine capsules. The minipigs were trained to eat these capsules. Thereafter, animals of both groups were allowed to drink water *ad libitum*. After 30 min, all animals were dosed by oral ingestion of gelatine capsules containing the proper amount of test substance [9-^14^C]-Oleic acid (10.8 μCi (0.4 MBq) [9-^14^C]-Oleic acid per kg bodyweight and 10 mg oleic acid per kg bodyweight) using a special dosing device and a wooden block preventing the animals to bite into the capsules. One animal of the control group A was inadequately dosed and was omitted from the experiment. The dosing capsules were prepared the day before the dose administration based on the bodyweight determined shortly before dosing and stored under nitrogen and protected from light at 2–10 °C. Immediately following dosing all animals received approximately 10 ml of water via a syringe. After 15 min, animals were allowed to drink water and eat their morning meal. This meal was eaten within a few minutes. The animals were housed in stainless steel metabolism cages under test conditions, 48 h prior to the experimental start date (dosing).

### Sampling schedule

Urine and faeces was collected at ambient temperature at 24 h time intervals, starting at 2 days prior to dosing and ending at 144 h post-dose. At each collection period, the cage was rinsed with 100 mL demi-water. At various defined time points blood samples of approximately 5 mL were taken via the cannula in the jugular vein into tubes with heparin as anticoagulant at pre-dose, 0.5, 1, 1.5, 2, 4, 6, 8, 12, 24, 36, 48, 60, 72, 96, 120 and 144 h post-dosing of [9-^14^C]-Oleic acid. The cannula was rinsed with physiological saline after each blood sampling. After the last blood sampling of the day the cannula was filled with 3 % poly vinyl pyrrolidone (PVP) and heparin, and closed with a lid. Subsamples of whole blood were collected for analysis and the remaining blood was used for preparation of plasma by centrifugation for 10 min at 2000 g. At the end of the experiment, the cages were rinsed thoroughly with water/ethanol/Triton X-100 (100/100/1; v/v/v). The minipigs were intravenously anaesthetized via the catheter with 60 mg sodium-pentobarbital per kg bodyweight. Next, the animals were sacrificed by exsanguination from the carotid artery. At sacrifice, the complete liver and contents of the gastrointestinal tract were collected. Prior to analysis of radioactivity, the faeces, liver and contents of the gastrointestinal tract were homogenized using an ultrathurrax.

### Analysis of radioactivity

Radioactivity in all samples was determined by liquid scintillation counting on a Tri-Carb 3100TR liquid scintillation counter using QuantaSmart™ software. All counts were converted to DPM using tSIE/AEC (transformed Spectral Index of external standards coupled to Automatic Efficiency Correction). Radioactivity in faeces, blood and liver tissue was determined by combustion analysis using a Packard Sample Oxidizer System 307. The CO_2_ formed was mixed with Carbosorb™ and Permafluor™ and measured by LSC.

### HPLC analysis for metabolic profiling

The HPLC system consisted of a Spectra Series P200 binary gradient pump from Thermo Separations and a Lablogic ß-RAM detector Model 4 in combination with Laura™ software and UV detection at 210 nm. HPLC analysis was achieved using a reversed phase BDS Hypersil C18 column (5 μ, 250 × 4.6 mm) eluted at a flow of 1 mL/min with 0.1 % formic acid in water as eluent A and 0.1 % formic acid in acetonitrile as eluent B. The flow of the scintillation fluid (Ultima-Flo™ M) was 3 ml/min. Radioactivity in faeces was extracted with acetonitrile before HPLC analysis. The residue was dissolved in DMSO : tetrahydrofuran (1:1; v:v) and was injected directly on the HPLC column.

### Pharmacokinetic and statistical analysis

Kinetic analysis of radioactivity (blood/plasma) was performed using WinNonlin® 6.3 applying non-compartmental model analysis. The following kinetic parameters were calculated where the data allow: Cmax, Tmax, half-life (t1/2), the volume of distribution, clearance, the Area under the concentration-time curve (AUC) (or AUC0-n, AUC0-∞) and the Mean Residence Time (MRT). Statistical analysis was assessed by using Mann–Whitney *U*-test (non-parametric test) using the Brightstat [34].

## Results

### Dose administration and animal observation

In order to investigate the influence of the polyglucosamine formoline L112 on the bioavailability of [^14^C]-oleic acid, female Göttingen minipigs were orally dosed without (group A) or with polyglucosamine formoline L112 (group B) followed by a single dose of [9-^14^C]-oleic acid.

The administered dose levels of oleic acid were between 9.78 and 10.0 mg/kg, which was close to the intended dose levels of 10 mg/kg bodyweight. The dosed amount of radioactivity was between 0.42 and 0.43 MBq/kg, which was closed to the intended dose of 0.4 MBq/kg bodyweight. Body weights were determined during acclimatisation, at the day of start acclimatisation to the metabolism cages and at sacrifice (Table 1). Weight loss was not an objective of the present study as the [^14^C]-oleic acid and formoline L112 was only given in a single dose to monitor effects of L112 on the bioavailability of [^14^C]-oleic acid.

**Table 1:** Bodyweights and dose levels of [9-^14^C]-oleic acid administered to untreated female Göttingen minipigs (group A - control) and female Göttingen minipigs that were pre-treated with 2 tablets of 500 mg formoline L112 (group B – formoline L112)

	Group A - control						Group B - formoline L112						
Animal number	A1	A2	A3	A4	A5	Mean ± SD	B1	B2	B3	B4	B5	B6	Mean ± SD
Body weight (kg):													
At acclimatisation	25.6	30.1	34.5	31.7	28.3	30.0 ± 3.4	32.2	30.0	31.4	30.5	33.9	31.4	31.6 ± 1.4
At dosing	26.0	27.7	31.9	31.3	27.6	28.9 ± 2.6	32.2	31.4	28.8	30.5	31.6	31.2	31.0 ± 1.2
At sacrifice	25.8	27.2	31.8	30.0	27.4	28.4 ± 2.4	32.9	29.6	28.2	28.2	30.4	30.0	29.9 ± 1.7
Dose (mg/kg)	9.78	9.89	9.92	9.92	9.89	9.88 ± 0.06	10.0	9.88	9.91	9.91	9.92	9.93	9.93 ± 0.04
Radioactivity (MBq/kg)	0.42	0.43	0.43	0.43	0.43	0.43 ± 0.00	0.43	0.43	0.43	0.43	0.43	0.43	0.43 ± 0.00

Values are presented as individual and mean ± standard deviation

The animals were checked for appearance and behaviour during acclimatisation, during treatment and at each sampling time. No study- or test substance-related signs of toxicity or unusual behaviour were observed. Formoline L112 did not cause any effect on faecal excretion; oily spotting, liquid stools, fecal urgency or incontinence, flatulence, and abdominal cramping, as frequently observed during anti-obesity treatment with an intestinal lipase inhibitor [5], was not observed in both treatment groups. No abnormalities were observed at autopsy.

### The influence of pre-treatment with the polyglucosamine formoline L112 on the pharmacokinetic behaviour of orally dosed [^14^C]-oleic acid in the Göttingen minipig

Following pre-treatment with or without formoline L112, minipigs were orally dosed with 10 mg [^14^C]-oleic acid/kg bodyweight. Blood samples were collected during a 144 h blood sampling period and radioactivity (representing [^14^C]-oleic acid and/or its metabolites) was determined in blood and plasma.

**Fig. 1:**
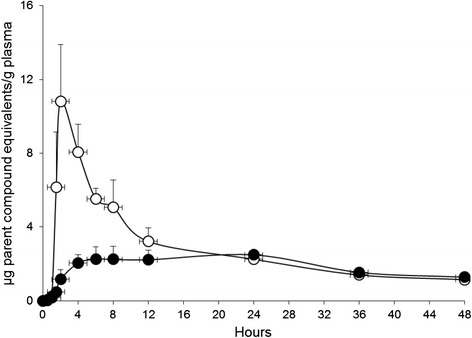
The mean time versus radioactivity concentration expressed as μg parent compound equivalents/g plasma of female Göttingen minipigs after a single oral dose of [9-^14^C]-oleic to untreated animals and animals that were pre-treated with formoline L112 between 0 and 48 h post-dose. The line with the open dots (○) (*N* = 5) represents the control group and the line with the filled dots (●) (*N* = 6) represents the formoline L112 pre-treated group. Error bars represent Standard Error of the Mean

Figure 1 represents the mean plasma concentrations of radioactivity (μg oleic acid equivalents and/or its metabolites/g plasma) versus time curve for the female minipigs after a single oral dose of 10 mg/kg [^14^C]-oleic acid to untreated control animals (group A) and animals pre-treated with formoline L112 (group B). The untreated animals of group A showed a clear peak of radioactivity (oleic acid equivalents and/or its metabolites) between 0 and 24 h, which declined considerably following pre-treatment with formoline L112.

**Fig. 2:**
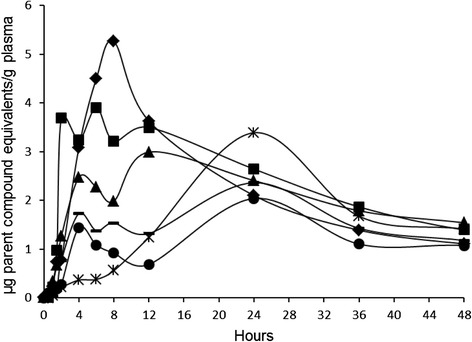
The individual time versus radioactivity concentration expressed as μg parent compound equivalents/g plasma of female Göttingen minipigs after a single oral dose of [9-^14^C]-oleic to animals that were pre-treated with formoline L112 between 0 and 48 h post-dose. The lines with the following markers ♦, ■, ▲, , ▬ and ● represent animal B1, B2, B3, B4, B5 and B6 of the formoline L112 pre-treated group, respectively

Figures 2 presents the individual time versus radioactivity concentration expressed as μg parent compound equivalents/g plasma of female Göttingen minipigs after a single oral dose of [9-^14^C]-oleic to animals that were pre-treated with formoline L112. In these plasma profiles a clear effect of a partial stomach emptying was observed around the time-points of feeding at start and end of the day, around 8 and 24 h post-dose. This effect was not observed for the untreated animals of group A. At the second emptying period of the stomach, the binding strength of formoline L112 for [^14^C]-oleic acid is clearly still present.

The plasma and blood kinetic data for [^14^C]-oleic acid (μg oleic acid equivalents and/or its metabolites/g) were analysed and were shown to best fit a non-compartmental model using the WinNonlin pharmacokinetic software program. The data could not be fitted to other kinetic models for both the untreated animals of group A and the formoline L112 treated animals of group B. The calculated pharmacokinetic parameters for [^14^C]-oleic acid of group A and group B are presented in Table 2 and are clearly affected upon treatment with formoline L112.

**Table 2:** Pharmacokinetic parameters (non-compartmental analysis) of [9-^14^C]-oleic acid and/or its metabolites in plasma and blood of Göttingen minipigs after a single oral dose of [9-^14^C]-Oleic to untreated minipigs (group A) and minipigs that were pre-treated with 2 tablets of 500 mg formoline L112 (group B)

Group	A	B	%^a^	A	B	%^a^
	Plasma			Blood		
Pharmacokinetic parameters						
T_max_ (h)	4 ± 3	16 ± 9*	-	4 ± 3	14 ± 11	-
C_max_ (μg/g)	14.1 ± 3.6	3.3 ± 1.2**	23.4	8.5 ± 2.2	2.1 ± 0.8**	24.7
AUC_(0-12h)_ (h* μg/g)	65.6 ± 6.0	21.6 ± 14.0**	32.9	40.0 ± 4.8	13.3 ± 9.1**	33.3
AUC_(0-24h)_ (h* μg/g)	98.4 ± 17.2	49.9 ± 20.8**	50.7	61.7 ± 13.1	29.8 ± 12.9**	48.3
AUC_(0-48h)_ (h* μg/g)	136 ± 28	91.0 ± 23.2*	66.9	88.3 ± 19.0	56.9 ± 13.1*	64.4
AUC_(0-60h)_ (h* μg/g)	148 ± 31	105 ± 25*	70.9	97.2 ± 20.6	67.3 ± 13.8*	69.2
AUC_(0-72h)_ (h* μg/g)	157 ± 32	116 ± 26	73.9	105 ± 22	76.4 ± 14.2*	72.8
AUC_all(0-144h)_ (h* μg/g)	190 ± 38	158 ± 32	83.2	139 ± 25	118 ± 16	84.9
Vz (g/kg)	7008 ± 2962	5629 ± 1946	-	8860 ± 1848	9515 ± 1647	-
Cl (g/h/kg)	40.7 ± 5.5	49.8 ± 8.3	-	48.4 ± 7.9	47.6 ± 7.3	-
MRTlast (h)	35.9 ± 1.8	50.2 ± 5.1**	-	43.6 ± 3.1	58.4 ± 5.7**	-

^a^Percentage of radioactivity remaining in plasma or blood following pre-treatment with formoline L112 compared to the untreated control group

* *P* ≤ 0.05, ** *P* ≤ 0.01

Upon treatment with formoline L112, the concentration of [^14^C]-oleic acid in plasma reaches its maximal value at a significantly later time-point namely 16 h post-dose compared to untreated animals, where the Tmax occurred at 4 h post-dose. The maximal concentration of [^14^C]-oleic acid in plasma decreases significantly from 14.1 μg/g to 3.3 μg/g upon treatment with formoline L112. In addition, upon treated with formoline L112 the internal exposure to [^14^C]-oleic acid as reflected by the area under the curve during the 0–12 h post-dose time interval (AUC_0-12h_), is significantly decreased to 32.9 % of the plasma value of [^14^C]-oleic acid in untreated animals. Even up to 24 h post-dose, the AUC_0-24h_ is significantly decreased to 50.7 % of the plasma value in untreated animals and this significant effect is prolonged up to 60 h post-dose. With regards to the concentrations of radioactivity in blood a similar pattern was observed as seen for plasma.

### Excretion of radioactivity

It was hypothesized that binding of [^14^C]-oleic acid to formoline L112 in the gastrointestinal tract would result in an increased excretion of radioactivity from the body via the faeces and a lower accumulation of radioactivity in liver of minipigs treated with formoline L112 (group B). In order to investigate the effect of formoline L112 treatment on the excretion of radioactivity, all animals receiving a single oral dose of [^14^C]-oleic acid were housed in metabolic cages suitable for the separate collection of urine and faeces at 24 h time intervals up to 144 h post-dose. Table 3 presents the recoveries of radioactivity in excreta (urine & faeces), liver and contents of the gastrointestinal tract at sacrifice of untreated (control) and formoline L112 pre-treated female Göttingen minipigs after a single oral dose of 10 mg/kg [^14^C]-Oleic acid.

**Table 3:** Summary table of the mean recovery of radioactivity ([9-^14^C]-oleic acid and/or its metabolites) in excreta (urine, faeces and cage wash), liver and contents of the gastrointestinal tract of Göttingen minipigs after a single oral dose of [9-^14^C]-oleic to untreated minipigs (group A) and minipigs that were pre-treated with 2 tablets of 500 mg formoline L112 (group B). Data are presented as the mean ± standard deviation

Sample	Recovery expressed as % of the applied dose	
	Group A	Group B
	Control (untreated)	Formoline l112
Urine		
-48–24 h	0.00 ± 0.01	0.00 ± 0.00
-24–0 h	0.01 ± 0.00	0.00 ± 0.00
0–24 h	0.79 ± 0.05	0.46 ± 0.28
24–48 h	0.28 ± 0.10	0.54 ± 0.14
48–72 h	0.09 ± 0.08	0.25 ± 0.17
72–96 h	0.08 ± 0.05	0.10 ± 0.04
96–120 h	0.05 ± 0.03	0.07 ± 0.04
120–144 h	0.03 ± 0.00	0.05 ± 0.02
Total	1.33 ± 0.20	1.46 ± 0.17
Faeces		
-48–24 h	0.00 ± 0.00	0.00 ± 0.00
-24–0 h	0.00 ± 0.00	0.00 ± 0.00
0–24 h	0.02 ± 0.02	0.06 ± 0.06
24–48 h	2.22 ± 2.05	1.45 ± 1.94
48–72 h	3.21 ± 2.93	3.02 ± 1.43
72–96 h	0.85 ± 0.83	2.17 ± 2.21
96–120 h	0.15 ± 0.13	0.32 ± 0.16
120–144 h	0.06 ± 0.03	0.13 ± 0.09
Total	6.51 ± 2.88	7.15 ± 2.87
Cagewash	0.07 ± 0.07	0.10 ± 0.07
Total excreted (sum urine, faeces, cagewash)	7.90 ± 3.13	8.72 ± 2.75
Liver at sacrifice (144 h post-dose)	0.37 ± 0.07	0.37 ± 0.03
GI-tract contents at sacrifice (144 h post-dose)	0.08 ± 0.04	0.13 ± 0.06

After 144 h post-dose only a part of the dosed radioactivity is excreted with urine 1.33 % and 1.46 % and faeces 6.51 and 7.15 %, for the untreated control animals of group A and formoline L112 treated animals of group B, respectively. At sacrifice the contents of the gastrointestinal tract contained 0.08 and 0.13 % of the dosed amount of radioactivity in group A and B, respectively. The liver at sacrifice contained a similar amount of radioactivity in both groups, namely 0.37 % of the dosed amount of radioactivity. Most likely, the majority of the dosed radioactivity is distributed into the body and/or further metabolized into CO_2_.

**Fig. 3:**
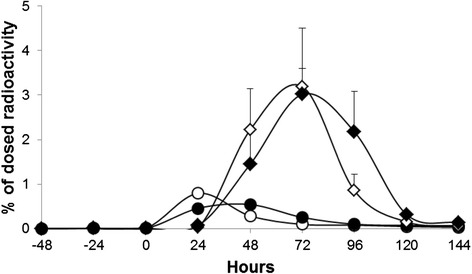
Excretion profile of radioactivity in urine and faeces of female Göttingen minipigs after a single oral dose of [9-^14^C]-Oleic to group A (control) and group B (minipigs pre-treated with a single oral dose of 2 tablets of 500 mg formoline L112 per animal). Open markers untreated, closed markers treated. Open dots ○, filled dots ● represent the excretion of radioactivity with urine, open triangles ◊, and filled triangles ♦ represent the excretion of radioactivity with faeces of group A and group B, respectively. Error bars represent Standard Error of the Mean

Although the standard deviations are relatively high, the excretion profile of radioactivity in urine and faeces seemed a little bit delayed in the formoline L112 pre-treated animals as compared to the untreated animals as presented in Fig. 3. The observation that treatment with formoline L112 did not affect the total excretion of radioactivity with faeces, but only seems to delay the excretion slightly, is in line with the pharmacokinetic data. The total area under the curve from start until the end of the study (AUC_0-144h_) was 190 ± 38 h.μg/g and decreased non-significantly to 158 ± 32 h.μg/g over the complete duration of the study. As animals only received one oral dose of formoline L112, the effects are most pronounced in the first period following dosing (0–24 h), but fade away after receiving additional oleic acid containing meals without formoline L112 treatment. Altogether, these observations indicate that formoline L112 reduces the bioavailability of [^14^C]-oleic acid between the 0–12 h, 0–24 h, 0–48 h and 0–60 h time intervals due to a delayed absorption and most interestingly prevents peak exposures of fatty acids in the body.

### Metabolite profiling of radioactivity in faeces

**Fig. 4:**
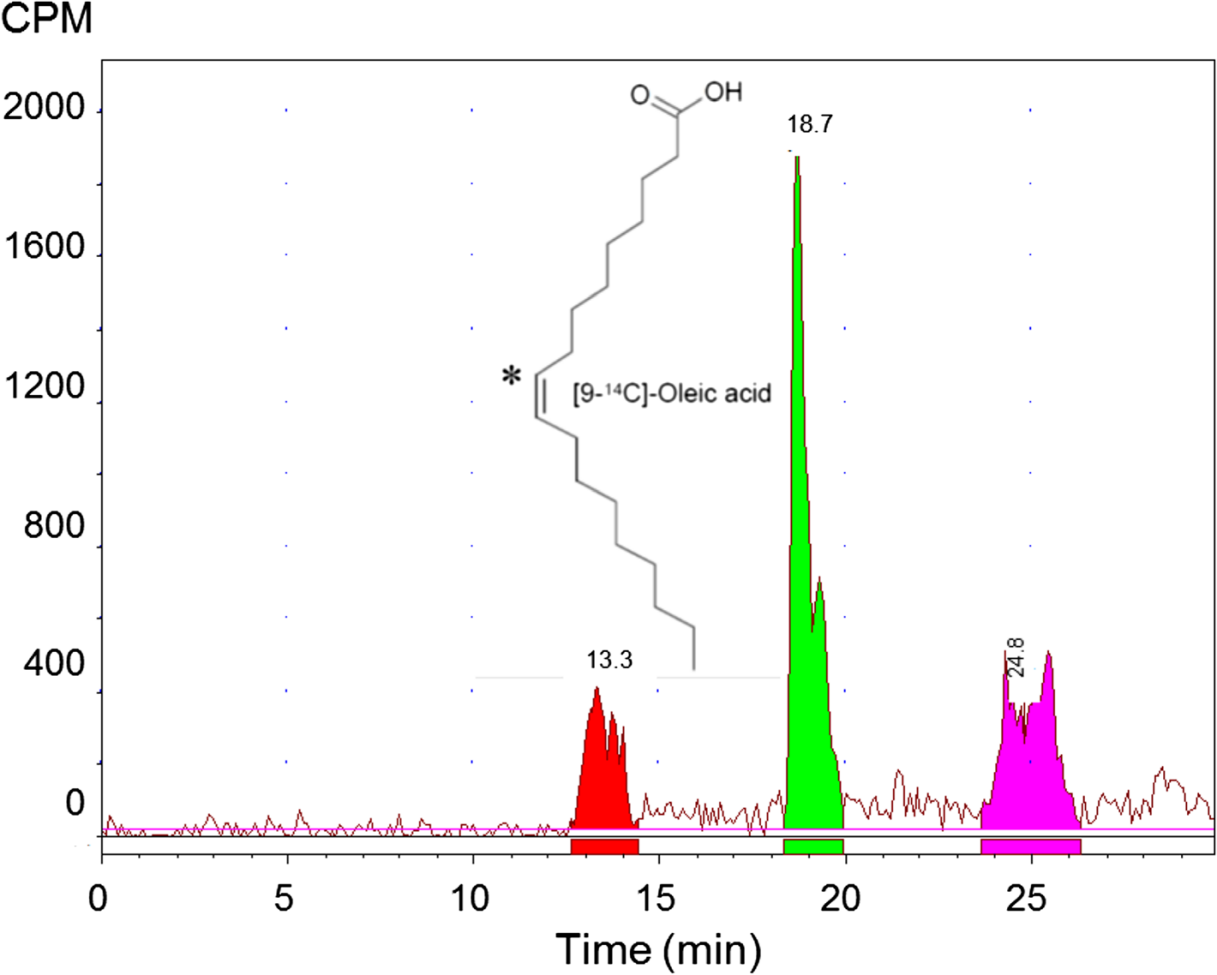
Representative radioactivity HPLC profile of a faeces extract sample. The peak eluting at a retention time of 18.7 min represents [9-^14^C]-oleic acid

Reverse phase HPLC radiometric analysis was used to investigate the profile of radioactivity in faeces for some selected samples of untreated and formoline L112 treated animals, and to separate and distinguish [^14^C]-oleic acid from its possible degradation products. The radioactivity was extracted from faeces and the mean recovery of excretion was 84.2 %. Figure 4 presents a representative radioactivity HPLC profile of faeces. The excreted radioactivity was eluted as 3 peaks, the first peak eluted at 12.1–14.7 min, the second peak eluted around 17.7–20.4 min ([^14^C]-oleic acid) and the third peak eluted at 23.4–24.7 min. There were no remarkable differences in metabolic profile between the untreated and formoline L112 treated animals.

### Balancing and recovery of radioactivity

**Fig. 5:**
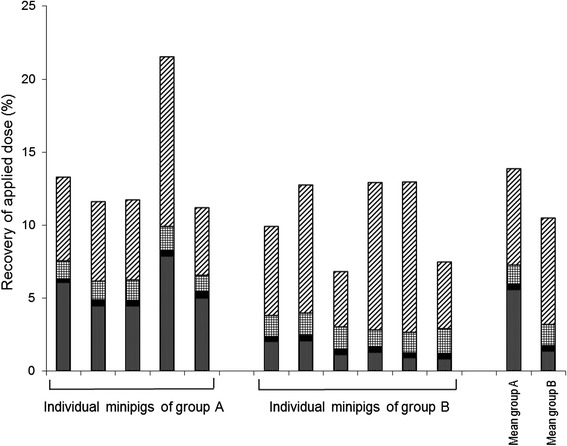
Individual and mean distribution and recovery of radioactivity in female Göttingen minipigs after a single oral dose of nominal 10 mg [9-^14^C-oleic acid/kg in group A (control) and group B (minipigs pre-treated with a single oral dose of 2 tablets of 500 mg formoline L112 per animal). Radioactivity is expressed as % parent compound. The following patterns , ,  and  represent the amount of radioactivity in blood (Cmax), liver, urine and faeces/gastrointestinal tract, respectively

A full balancing of the administered radioactivity was not given as the volatile matter was not trapped during the test and as all organs, tissues and residual carcasses of the minipigs were not collected and analysed for radioactivity. A recovery and balance of the non-volatile matter of collected samples is shown in Fig. 5. Here a relevant difference between the untreated control group A and the formoline L112 treated group B is clearly visible. As this study was not a mass-balance study because of the abovementioned reason, the overall recovery was 34.7 and 17.9 % (based on the AUC_0-12h_) and 13.9 and 10.6 % (based on the C_max_) of the dosed amount of radioactivity in the untreated and formoline L112 treated minipigs, respectively.

## Discussion

The current strategies to reduce the burden of obesity and its consequences include lifestyle intervention (diet/physical activity), pharmacotherapy, and bariatric surgery.

At present, because of dissatisfaction with high costs and potentially hazardous and/or embarrassing side-effects of the current available therapies, the potential of natural products like polyglucosamines for treating obesity is discussed, and may serve an excellent alternative strategy for treating obesity [3, 7]. Polyglucosamines have been promoted as a fiber of natural origin that may help reduce the absorption of fat due to its ability to “bind” fat and bile salts in the human gastrointestinal tract and pass this complexed fat-polyglucosamine mixture out of the body. This action reduces absorbance of dietary fat, and has prompted the use of these substances to help with weight loss [7]. The objective of this study was to provide data on the influence of formoline L112 on the bioavailability of [9-^14^C]-oleic acid as a model compound in the adult female Göttingen minipig. Upon treatment with formoline L112, the T_max_ of [^14^C]-oleic acid in plasma was shifted from 4 to 16 h. The C_max_ decreased significantly from 14.1 μg/g to 3.3 μg/g upon treatment with formoline L112. In addition, upon treated with formoline L112 the internal exposure to [^14^C]-oleic acid as reflected by the area under the curve during the 0–12 h post-dose time interval (AUC_0-12h_), is significantly decreased to 32.9 % of the plasma value of [^14^C]-oleic acid in untreated animals. Even up to 24 h post-dose, the AUC_0-24h_ is significantly decreased to 50.7 % of the plasma value in untreated animals and this significant effect is prolonged up to 60 h post-dose. In the formoline L112 pretreated minipigs, a clear effect of a partial stomach emptying was observed around the time-points of feeding at start and end of the day, around 8 and 24 h post-dose. This effect was not observed for the untreated animals of group A. At the second emptying period of the stomach, the binding strength of formoline L112 for [^14^C]-oleic acid is clearly still present.

In untreated subjects, oleic acid is absorbed practically completely and is distributed in the organism after oral administration [35, 36]. Breakdown via the pathways of lipid metabolism takes place by successive β-oxidation and excretion as CO_2_. Like other free fatty acids, oleic acid is absorbed by the intestinal walls where it is converted to triglycerides. The triglycerides are transported in lipoproteins in serum or in chylomicrons in the lymphatic vessels and are stored in adipose tissue or metabolized. Thirtyone test persons with normal fat assimilation were given oral doses of radioactively labelled triolein and oleic acid; after 6 days they had excreted less than 10 % of the substances in the faeces [37]. This pattern is similar as has been observed in the present study.

Considerable evidence suggests that exposure to increased free fatty acid concentrations plays a key role in the pathogenesis of insulin resistance. Free fatty acids cause both insulin resistance and inflammation in the major insulin target tissues (skeletal muscle, liver and endothelial cells) and thus are an important link between obesity, insulin resistance, inflammation and the development of type 2 diabetis, hypertension, dyslipidemia, disorders of coagulation and atherosclerotic vascular disease [38]. Reducing plasma FFA concentration in obese and type 2 diabetic (T2DM) subjects improves insulin sensitivity [39].

Endotoxin, the major constituent of the outer cell membrane of gram negative bacteria, crosses the gut mucosal membrane to enter the circulation and directly stimulates inflammatory pathways. High-fat meals result in an increase in endotoxin levels in animal and human studies, with a greater increase in metabolic disease states [40–42]. A high-fat diet constitutes one of the major risks for developing obesity, diabetes, atherosclerosis, and other feeding-related disorders. Consumption of a high fat diet induces inflammation in the adipose tissue, liver, and skeletal muscle by increasing endotoxin lipopolysaccharide levels in the intestinal lumen, as well as by enhancing LPS action via Toll-like receptor 4. This inflammation plays a critical role in the development of obesity and insulin resistance [41, 43].

In the present study it has been demonstrated that formoline L112, which is used for weight management, weight loss and to lower cholesterol blood levels, decreases peak levels of [^14^C]-oleic acid in plasma and also reduces the overall internal exposure to [^14^C]-oleic acid. Treatment with formoline L112 reduces (as judged by C_max_ & AUC) and delays (as judged by T_max_) fat absorption from the gastrointestinal tract into the systemic circulation and limits peak exposure to free fatty acids which may contribute to a more beneficial condition in overweight humans.

According to the product information of formoline L112, two tablets of 500 mg should be taken twice daily with two main meals with the highest fat content, for instance at breakfast and dinner. In the present study, minipigs were only dosed once with formoline and did not receive formoline L112 at the second meal on the dosing day, thus it is expected that a second dose would even reduce uptake to a higher extent.

In vitro tests performed by the supplier of the polyglucosamine with a synthetic mixture of dietary oils and lipids as model oil indicated no preference or discrimination of any fatty acid for interaction with formoline L112 (unpublished).

Therefore, it is assumed that the effects of polyglucosamine on the bioavailability of [^14^C]-oleic acid are transferable to other fats.

## Conclusion

In conclusion, treatment with the polyglucosamine formoline L112 reduces (as judged by C_max_ & AUC) and delays (as judged by T_max_) fat absorption from the gastrointestinal tract into the systemic circulation and limits peak exposure to free fatty acids which may contribute to a more beneficial condition in overweight humans.

## Abbreviations

AUC: area under the curve
Cl: clearance
Cmax: maximum concentration in plasma or blood
DPM: desintegrations per minute
EMA: European Medicines Agency
FDA: food and drug administration
FFA: free fatty acids
GABA: gamma amino butyric acid
GIT: gastrointestinal tract
GRAS: Generally Recognized As Safe
HPLC: high pressure liquid chromatography
LPS: lipopolysaccharide
LSC: liquid scintillation counting
MBq: megabecquerel
MRT: Mean Residence Time
SD: standard deviation
T2DM: type 2 diabetes mellitus
Tmax: time after dosing associated with the maximum observed concentration in plasma or blood
UV: ultraviolet
Vz: volume of distribution
